# 

**Published:** 1896-10

**Authors:** 


					﻿Southern Medicai Record.
A Monthly Journal of Medicine and Surgery.
Vol. XXVI.	ATLANTA, GA., OCTOBER, 1896.	No. X>
BERNARD WOLFF, M. D., Editor.
LOUIS H. JONES, M. D., Business Manager.
Associate Editors :
I A. W. GRIGGS, M. D.
WILLIS F. WESTMORELAND. M. D.
j. McFadden gastón, m. d.
TERMS: $1.00 Per Annum; Single Copy, 10Cents.	Contents on Page 6.
Entered at the Post-Office at Atlanta, Ga., as Second-Class Matter.
The Foote & Davies Co., Atlanta, Ga.
				

